# Structure, signal transduction, activation, and inhibition of integrin αIIbβ3

**DOI:** 10.1186/s12959-023-00463-w

**Published:** 2023-02-13

**Authors:** Honglei Xin, Jiansong Huang, Zhiqun Song, Jianhua Mao, Xiaodong Xi, Xiaofeng Shi

**Affiliations:** 1 Department of Hematology, Second Affiliated Hospital of Nanjing Medical University, Nanjing, Jiangsu 210003 China; 2 Department of Hematology, The First Affiliated Hospital, Zhejiang University School of Medicine, Zhejiang, Hangzhou 310003 China; 3 Shanghai Institute of Hematology, State Key Laboratory of Medical Genomics, Collaborative Innovation Center of Hematology, Ruijin Hospital, Shanghai Jiao Tong University School of Medicine, Shanghai, 200025 China; 4 Jiangsu Province People’s Hospital and Nanjing Medical University First Affiliated Hospital, Nanjing, Jiangsu 210029 China

**Keywords:** Platelet activation, Integrins, αIIbβ3, Hemostasis, Thrombosis

## Abstract

Integrins are heterodimeric receptors comprising α and β subunits. They are expressed on the cell surface and play key roles in cell adhesion, migration, and growth. Several types of integrins are expressed on the platelets, including αvβ3, αIIbβ3, α2β1, α5β1, and α6β1. Among these, physically αIIbβ3 is exclusively expressed on the platelet surface and their precursor cells, megakaryocytes. αIIbβ3 adopts at least three conformations: i) bent-closed, ii) extended-closed, and iii) extended–open. The transition from conformation i) to iii) occurs when αIIbβ3 is activated by stimulants. Conformation iii) possesses a high ligand affinity, which triggers integrin clustering and platelet aggregation. Platelets are indispensable for maintaining vascular system integrity and preventing bleeding. However, excessive platelet activation can result in myocardial infarction (MI) and stroke. Therefore, finding a novel strategy to stop bleeding without accelerating the risk of thrombosis is important. Regulation of αIIbβ3 activation is vital for this strategy. There are a large number of molecules that facilitate or inhibit αIIbβ3 activation. The interference of these molecules can accurately control the balance between hemostasis and thrombosis. This review describes the structure and signal transduction of αIIbβ3, summarizes the molecules that directly or indirectly affect integrin αIIbβ3 activation, and discusses some novel antiαIIbβ3 drugs. This will advance our understanding of the activation of αIIbβ3 and its essential role in platelet function and tumor development.

## Introduction

**Fig. 1:**
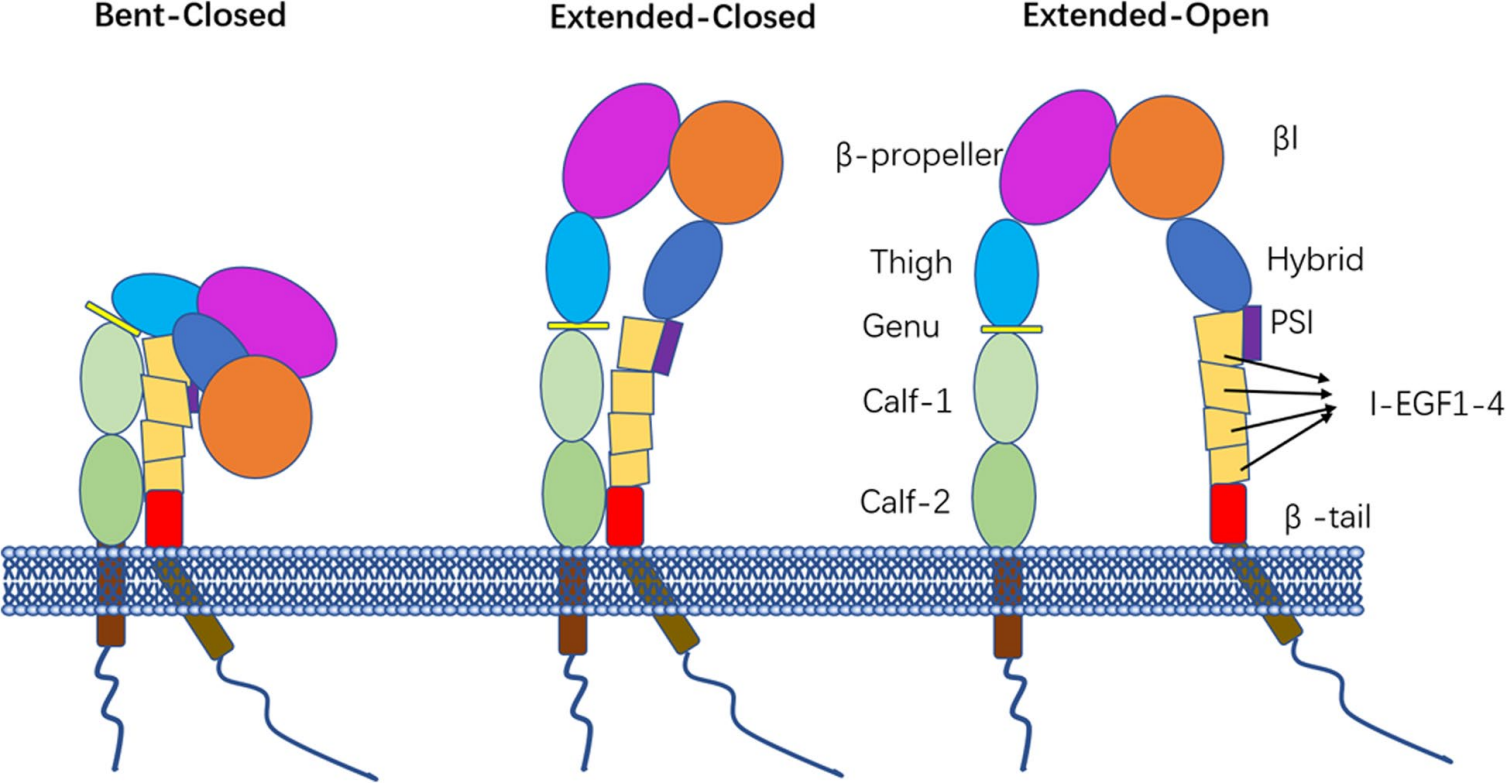
The structure of integrin. The conformational changes from bent-closed, to extended-closed, and finally to extended-open state [3, 4]

Integrins are key mediators of cell-matrix and cell-cell adhesion in physiology and disease. Recently, the 2022 Albert Lasker Basic Medical Research Award was presented to three scientists for their discoveries concerning the integrins [1]. Integrins are a large family of cell surface adhesion receptors. They comprise heterodimeric transmembrane glycoprotein complexes assembled from noncovalently bound α and β subunits. Every integrin subunit contains a large extracellular domain, transmembrane domain, and short cytoplasmic domain [2]. The α subunit comprises a β-propeller, followed by thigh, genu, calf-1, and calf-2 domains. Some α-subunits have an inserted domain (αI domain), serving as a major ligand-binding site [3]. The β subunit includes a βI domain, a hybrid domain, a plexin-sempahorin-integrin (PSI) domain, four cysteine-rich epidermal growth factor (EGF) repeats, and a membrane-proximal β-tail domain (βTD). Integrins adopt at least three conformations: bent-closed (resting state), extended-closed (intermediate state), and extended-open (activated state) [3, 4] (Fig. 1). The α and β cytoplasmic domains are in close proximity to each other in the resting state. The separation of the two tails is triggered by signal transmission from the intracellular domain to the extracellular domain. This is called ‘inside-out signaling’, which is stimulated by agonists, converts integrin into an extended conformation and increases the affinity of αIIbβ3 for ligands containing a common Arg-Gly-Asp (RGD) motif [5]. Subsequently, ligand binding to integrin initiates the outside-in signaling pathway. This triggers and amplifies various cellular events, including cell spreading, stable thrombus formation, and clot retraction. Transmembrane domain of integrin is involved in bidirectional signaling. Once integrin is activated, the transmembrane domain separates and serves as a signal transmitter. Transmembrane domain of αIIbβ3 is most stably associated, when compared to that of αvβ3 and α5β 1[6]. Truncated integrins lacking the transmembrane domain and cytoplastic domain are constitutively active [7], while αvβ3 ectodomain without transmembrane domain is in the resting bent conformation.

Integrin αIIbβ3 is the most abundant receptor expressed on the platelet surface [8]. It binds fibrinogen, which is central to platelet aggregation and spreading. The hemostasis process occurs as follows: vessel wall injury results in collagen exposure, which is bound by von Willebrand factor (vWF) and results in platelet recruitment to the site of the damaged vessel wall. vWF mediates the linkage between platelets and collagen via receptors on the platelet membrane [9]. Platelets are activated by modification from a disc shape into a dendritic form. Activated platelets release several prothrombotic molecules such as adenosine diphosphate (ADP). ADP triggers platelet aggregation, thus recruits more platelets to the injury site. Moreover, activated platelets can trigger the coagulation cascade, resulting in the full activation of platelets, which contribute to the formation of solid clots. αIIbβ3 has become a validated antithrombotic target since it is the main integrin in platelets. This review focuses on the role of integrin αIIbβ3 in platelet activation, which lays the foundation for our understanding of antiplatelet and antitumor therapy.

### Structure of αIIbβ3

Integrin αIIbβ3 contains extracellular-, transmembrane-, and cytoplasmic domains. The transmembrane segments of the α- and β-subunits are responsible for signaling events [1]. The αIIb transmembrane segment is a linear α spiral perpendicular to the cell membrane, followed by a corner region of the main chain, whereas β3 is a linear α-helix at an angle of 25° to αIIb [10]. Moreover, there is a conserved membrane-proximal GFFKR region in the αIIb and HDRxE region in the β3 tail, which form a “clasp” and contribute to maintaining an inactive state of αIIbβ3 [11]. Interactions between the transmembrane domains of αIIb and β3 are vital for maintaining integrin in the resting state. The first clasp is called the “outer membrane clasp” (OMC) and is formed by the interaction of the αIIb subunit (Gly^972^ and Gly^976^) with the β3 subunit (Gly^708^) near the outer membrane of the transmembrane segment of integrin in the lipid bilayer. The second clasp is termed the “inner membrane clasp” (IMC) and is assembled from a hydrophobic region of αIIb (F^992^-F^993^) and electrostatic αIIb salt bridge (R^995^)/β3 (D^723^) located near the inner membrane of the transmembrane segment of the integrin. Mutations in the IMC and the OMC can disturb the interaction of the transmembrane domain. The electrostatic interaction between R^995^ in αIIb and D^723^ in β3 is important for maintaining IMC stability, thus stabilizing the low-affinity state and regulating integrin activation [12]. Artificial dimerization or disulfide binding can inhibit integrin activity, whereas the R^995^ mutation in αIIb or D^723^ mutation in β3 can stretch and activate integrins [13]. The β3 cytoplasmic segment is longer than αIIb and is more conserved than αIIb. Furthermore, β3 plays an important role in integrin signal transduction. Each extracellular domain of the integrin is composed of a headpiece and a leg piece. In the resting state, the headpiece folds onto the leg piece such that the conformation has a low affinity for the ligands. Once stimulated, the αIIbβ3 headpieces are exposed to ligands such as vWF, fibrinogen, fibronectin, and vitronectin [3]. The swing-out motion of the hybrid domain and switchblade-like movement of the headpiece from the leg domains are the two major conformational rearrangements of αIIbβ3 [14]. A mutation of L^33^ to P^33^ in β3 makes the PSI-, I-EGF-1-, and I-EGF-2 domains more flexible, thus improving the ability of β3 to bind fibrinogen and increasing the risk of thrombus formation [15]. However, there are still controversial results. Zhou et al. designed a chimeric β3 with both L^33^ and P^33^ forms and discovered that L^33^ to P^33^ does not cause conformational changes or elevated ligand binding in β3 integrin. A full-length β3 fragment that adopts intermediate and fully extended conformations of β3 was also designed to provide high-resolution information on how β3 integrin extends at its PSI-, I-EGF1-, and I-EGF2 domains (β-knee region) [16]. Moreover, in platelets with β3 P^33^ mutation, the Src and FAK are highly activated, especially at high shear rates and β3 P^33^ mutation knockin mice show increased platelet adhesion, aggregation, and clot formation [17, 18]. They also demonstrated that the I-EGF2 C1-C2 loop plays a critical role in integrin extension, which may be an excellent target for regulating integrin function [19].

### Interaction of β3 with signaling factors

#### Interaction of β3 NPxY^747^ motif with Talin

**Fig. 2:**
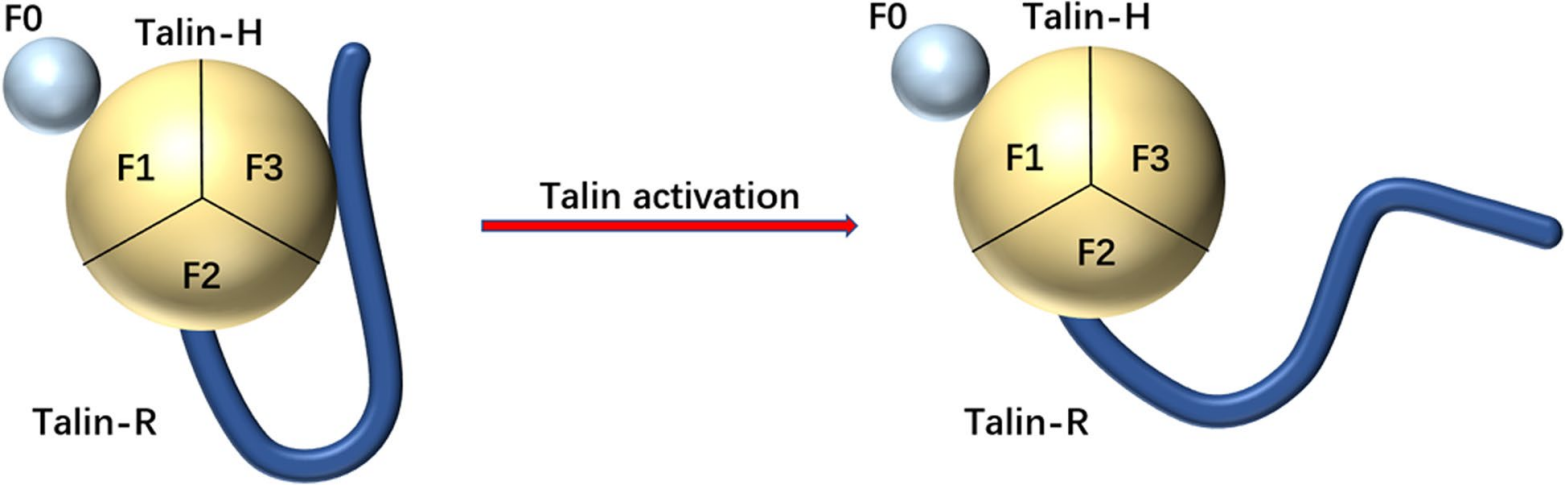
The structural change of talin from the inactive form to the active form [21]

Talin is a cytoskeletal protein that consists of an N-terminal head domain (talin-H) and C-terminal flexible rod domain (talin-R). Talin-H includes an F0 domain and an FERM domain (band 4.1, ezrin, radixin, moesin), which contains three subdomains: F1, F2, and F3 [20, 21] (Fig. 2). F3 resembles a phosphotyrosine-binding domain and is responsible for binding the membrane-proximal NPxY motif of β3, phosphatidyl inositol phosphate kinase type 1γ (PIPK1γ), and layilin [22]. Talin adopts a dual auto-inhibited conformation in the resting state. Talin-R masks the β3 tail binding sites in the F3 domain. Meanwhile, talin-R is negatively charged and repels the talin-F2F3/talin-R complex from the membrane. Furthermore, phosphatidylinositol 4,5-bis-phosphate (PIP2)-enriched membranes contribute to talin activation. The strong affinity of PIP2 for positively charged talin-F2F3 pulls talin-F2F3 domains to the membrane. Meanwhile, talin-R is pushed away; this is called the pull-push model [21]. There are two integrin binding sites (IBS) on talin: one is located in talin-H (IBS1), while the other is in talin-R (IBS2). Binding of talin to integrin tails is the final and most important step leading to integrin activation; therefore, its activity should be well regulated [23]. Interactions between the talin-F3 domain and β3 tail can disrupt the salt bridge formed by R^995^ in αIIb and D^723^ in β3, resulting in conformational changes that increase fibrinogen affinity. During this process, the talin-F3 domain interacts with PIPKIγ to catalyze PIP2 production, with talin relieving its auto-inhibition between the FERM domain and the C-terminal rod domain to become activated [24]. Gα13 switch region 2 competes with talin-R and directly binds to talin-F3, thus relieving its autoinhibition and activating talin [25]. Furthermore, talin-F3 binds to the highly conserved NPxY^747^ motif of β3. The loop structure in talin- (309–405) interacts with the α-helix in the membrane-proximal cytoplasmic domain of β3 which causes rearrangement of the transmembrane segment and interferes with D^723^; this disrupts the salt bridge resulting in αIIbβ3 activation [26]. Mutation of the NPxY^747^ motif in β3 and mutation of talin-F3 can impair talin binding and reduce integrin affinity. Talin-R includes several vinculin-binding sites (VBSs) and several actin-binding sites that connect to the extracellular matrix (ECM). Furthermore, full-length vinculin is autoinhibited unless activated, which is similar to mode of operation of talin. Activated talin binds to vinculin via the two vinculin-binding sites (VBSs) within the talin R3 helical bundle. This activates vinculin by disrupting the vinculin head-tail interaction and directly links it to the ECM.

Recent findings suggested that the Rap1/talin-1 (Talin includes talin1 and talin2) interaction is vital for platelet integrin activity. Rap1a and Rap1b are Ras subfamily members which share a high degree of similarity in vertebrates [27]. Rap1a and Rap1b may have some overlapping functions [4]. Rap1b deficient mice show impaired platelet function. Rap1-GTP-interacting adaptor molecule (RIAM) was previously considered a Rap1 effector [28]. Talin binding sites to RIAM are identified in R3, and R8 domains and F3 of talin [29]. However, increasing studies indicate that the *RIAM* gene in mice is dispensable for platelet integrin functions [30, 31]. RIAM depleted mice exhibit severe leukocytosis and impaired leukocyte extravasation, suggesting that *RIAM* may be a new target for human inflammatory diseases [32]. Furthermore, Rap1 directly interacts with talin1 and enhances talin-induced integrin activation [29]. Platelet functions such as aggregation, spreading, hemostasis, and thrombosis are impaired in Rap1 binding-deficient talin1 knock-in mice [33]. There are two identified talin1 binding sites to Rap1: one is located in the F0 domain and the other is in the F1 domain. Rap1 binding to the F1 domain makes a greater contribution to αIIbβ3 activation than binding to the F0 domain [4]. αIIbβ3 activation requires the talin1 F1/Rap1 interaction whereas the talin 1 F0/Rap1 interaction is not essential [34].

#### Interaction of the β3 NxxY^759^ motif with kindlin

Kindlins, similarly as talin, have important implications for integrin signal transduction. Kindlin is named after the Kindler syndrome, a rare skin blister disease characterized by blistering, skin atrophy, photosensitivity, pigmentation defects and so on [35]. All these symptoms are related to cell adhesion defects [36]. Members of the kindlin family include kindlin-1, kindlin-2, and kindlin-3, and they exhibit a high degree of similarity [37]. Kindlin-1 is mainly expressed in epithelial cells [38]. Kindlin-2 is ubiquitously expressed in tissues and is mainly found in skeletal- and smooth muscle cells. Knockout of kindlin-2 results in mouse embryonic lethality [39, 40]. A recent study reported that kindlin-2 depletion induces cardiac myocyte hypertrophy and increases the heart weight [41]. Surprisingly, kindlin-2 may play a central role in the occurrence and development of fatty liver and diabetes mellitus [42, 43], suggesting that it may be involved in the metabolism of blood sugars and lipids. Additionally, kindlin-2 binds to integrin-linked kinase (ILK) and promotes focal adhesions [44, 45]. Kindlin-3 was previously thought to be restricted to hematopoietic cells; however, more recent work shows that is also present in endothelial cells [39]. Kindlin-3-deficient leukocytes fail to arrest and extravasate into inflammatory tissues which causes leukocyte adhesion deficiency syndrome type III; this manifests in massive bleeding and recurrent infection [46]. Patients with kindlin-3 gene variants present with impaired platelet function, lymphocytosis and granulocytosis [47].

Kindlins and talins are FERM-containing proteins. Moreover, kindlin comprises a loop region in the F1 domain and a pleckstrin homology (PH) domain inserted in the F2 domain [48]. Unlike the extended structure of talin, kindlin adopts a cloverleaf structure [49] and multiple salt bridges in the F1 and F3 subdomains stabilize the cloverleaf-like FERM domain which supports integrin αIIbβ3 activation [49]. Mutations of interface residues in the F3 subdomain impair the ability of kindlin to support integrin αIIbβ3 activation _[_49_]__._ The binding of kindlin to the cytoplasmic tail of β3 integrin cooperates with talin to activate β3. The β3 membrane-proximal NxxP^747^ motif is the binding site for talin, while the membrane-distal NxxY^759^ motif is the binding site for kindlin. Both talin and kindlin are necessary for regulating integrin affinity; however, the mechanism of cooperative activation remains unclear [50]. One possible mechanism involves kindlin binding to the β3 tail to expose and stabilize the proximal talin-binding site in β3, resulting in talin destroying the αIIbβ3 transmembrane domain and activating αIIbβ3. Alternatively, talin binds to β3, followed by the recruitment of kindlin to trigger integrin αIIbβ3 clustering, and enhances its affinity for extracellular ligands [51].

Many proteins have been identified to interact with kindlin, including paxillin, adhesion and degranulation promoting adaptor protein (ADAP), migfilin, α-actinin, Src, and F-actin. In recent years, paxillin was thought to interact with kindlin-3 via its F0 subdomain [52], while other studies suggest that binding occurs through its F1- and PH domain [51, 53]. Therefore, the precise mechanism underlying the interaction between paxillin and kindlin-3 remains unclear.

#### Interaction of β3 R^760^GT^762^ with Src family kinase (SFK)

Src binds to the last three amino acid residues in the β3 cytoplasmic tail (R^760^, G^761^, and T^762^) via its SH3 domain in platelets mainly mediating outside-in signaling transduction [54]. The platelets with R^760^GT^762^-deleted β3 show impaired spreading on immobilized fibrinogen, but intact soluble fibrinogen binding [55, 56]. Src contains the following domains: an N-terminal 14-carbon myristoyl group, two highly conserved Src homology domains (SH2 and SH3), a catalytic domain, and a C-terminal tail [57]. Recently our group found that the residues, especially E^97^, in the RT loop of Src SH3 are critical for interacting with β3 [58]. DCDBS84, a small molecule disrupting β3/Src interaction, inhibits the outside-in signaling-regulated platelet functions [58]. Src myristoylation promotes cell membrane binding and is imperative for function in vivo. The SH2 region of Src interacts with its C-terminal phosphorylated Y^529^, which is maintained by the c-Src kinase, Csk. Csk also binds to the β3 cytoplasmic tail. Once αIIbβ3 binds to fibrinogen, Src Y^529^ is dephosphorylated and Y^418^ is phosphorylated [59]. This is followed by Csk dissociation from β3, and the activation of Src triggers a tyrosine phosphorylation cascade leading to phosphorylation of the β3 tail at Y^747^ and Y^759^ [60, 61]. Src can interact with FAK (focal adhesion kinase) to form a complex and mediates cell growth and migration. In addition, tumor growth and metastasis are associated with Src-FAK complex too [62]. Spleen tyrosine kinase (Syk) is downstream of Src and implicated in outside-in signaling of αIIbβ3. Platelets in Syk^−/−^ mice show impaired spreading [59]. Moreover, the cleavage of the β3 Src-binding site by calpain is regarded as a molecular switch between cell spreading and retraction [63]. Loss of Src binding to the β3 tail results in defective spreading and enhanced clot retraction mediated by RhoA, indicating that Src may impose restrictions on RhoA [63]. There are four types of SFKs in mouse platelets: Src, Fgr, Fyn, and Lyn. Each plays a different role in platelet activation. Mice deficient in SFK are characterized by impaired tyrosine phosphorylation [61]. However, individual depletion of each type does not result in severe bleeding, indicating that overlapping functions may exist in the four SFKs [64]. Fyn binds to the β3^721–725^ domain (I^721^HDRK^725^). Fyn^−/−^ mice show prolonged bleeding and delayed platelet spreading [65]. The β3^721–725^ domain is also required for binding of other proteins, including focal adhesion kinase, skelemin, and paxillin. The β3^721–725^ domain is close to the salt bridge; therefore, mutations that affect the stability of the transmembrane domain induce conformational changes. Junctional adhesion molecule A (JAM-A) is associated with the regulation of platelet function by inhibiting the recruitment of c-Src kinase, which inhibits c-Src activation and outside-in signaling [66]. JAM-A-knockout mice have shorter bleeding times and enhanced thrombosis capability [67].

#### Interaction E^731^XE^733^ of β3 with Gα13

Gα13 is a heterotrimeric guanine nucleotide-binding protein and can interact with E^731^XE^733^ motif in β3 tail. αIIbβ3-expressing CHO cells or platelets interfered with Gα13 siRNA neither spread well on fibrinogen-coated surface nor activate Src [68]. While interference of Gα13 stimulates the small guanosine triphosphatase RhoA with accelerated cell retraction [68]. Platelets treated with the myristoylated EXE-motif-containing peptide, which disrupts Gα13/β3 E^731^XE^733^ interaction, demonstrate poor spreading and aggregation, while strong clot retraction [69].

**Fig. 3:**
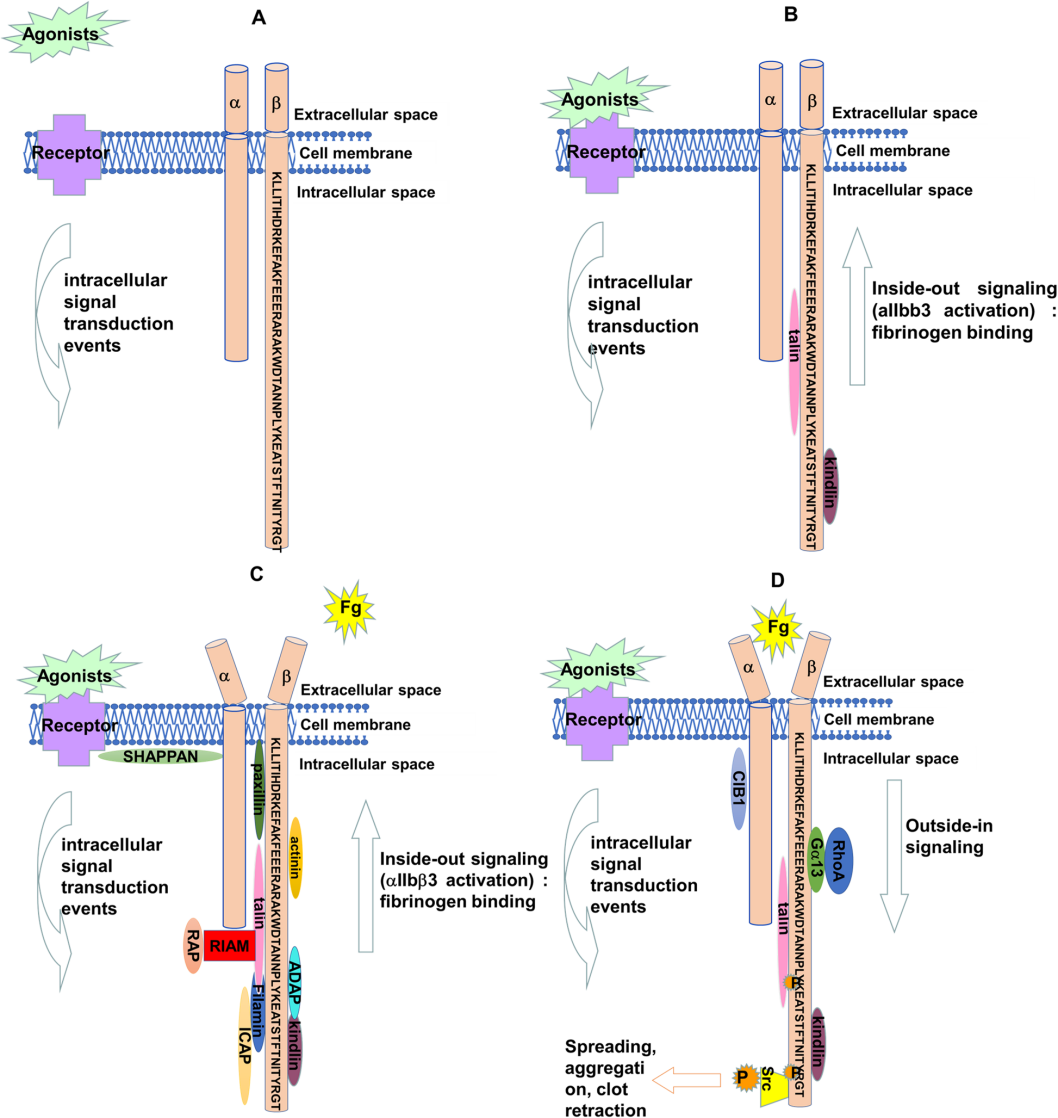
αIIbβ3 bidirectional signaling. The agonists, such as thrombin, ADP, epinephrine, 5-HT, TXA2, or collagen, bind to their receptors causing intracellular signaling events, which then lead talin to bind to β3 tail with the help of kindlin (A and B). Then, αIIbβ3 conformation changes from bent-closed to extended-open state. Thus, the affinity of αIIbβ3 to fibrinogen increases (C). This process is called inside-out signaling transduction (C). Once fibrinogen binds to αIIbβ3, signal transfers from outside to inside, named as outside-in signaling transduction, causing Gα13 to bind to E^731^XE^733^ motif, and Src to bind to R^760^GT^762^ motif, accompanied by phosphorylation of Y^747^ and Y^759^ in β3 tail, as well as phosphorylation of Y^416^ in Src. At last, the platelet spreading, aggregation, and clot retraction develop (D). For representative purposes, β tail is outsized

The αIIbβ3 bidirectional signaling and the interactions are shown in Fig. 3.

#### Other factors involved in αIIbβ3 bidirectional signaling

##### Proteins which promote αIIbβ3 activation

Phosphoinositide 3-kinases (PI3Ks) are a group of lipid kinases, including different isoforms [70]. PI3Kα is involved in the outside-in signaling of vWF-engaged αIIbβ3 integrin. An absence or inhibition of PI3Kα results in a decreased thrombus size after superficial injuries of mouse mesenteric arteries and an increased time to arterial occlusion after carotid lesion, without prolonged tail bleeding time [71]. PI3Kβ is responsible for PIP2 production and is linked to the activation of Rap1b and serine/threonine kinase [72]. Inhibition of PI3Kβ or PI3Kγ leads to a profound defect in platelet aggregation, hemostatic plug formation, and arterial thrombosis [72].

Paxillin is a key focal adhesion adaptor/scaffold protein which binds with the F0 subdomain of kindlin-1 or the PH domain of kindlin-2 and kindlin-3 to support αIIbβ3 activation [73]. Interaction between paxillin and the kindlin-2 F0 domain contributes to the recruitment of paxillin to focal adhesions which leads to cell migration and transmission of mechanical force along the cytoskeleton [74]. Recently, it is found that the interaction between paxillin and PH domain of kindlin-3 plays an important role in supporting integrin αIIbβ3 outside-in signaling in platelets [53]. Furthermore, co-existence of paxillin and kindlin-1 greatly improves binding of the talin-H to β3, suggesting that paxillin may facilitate the interaction between talin and β3. Suppression of paxillin expression significantly inhibits αIIbβ3 activation [52]. Moreover, paxillin binds vinculin and talin, leading to talin and vinculin recruitment into nascent adhesions and inducing focal adhesion maturation [75]. Besides the indirect interaction, paxillin possibly directly interacts with talin and β3 integrin. It functions as a signaling molecule, recruiting other focal adhesion proteins, modulating F-actin polymerization, and thus generating feedback signaling [76].

Integrin-linked kinase (ILK) is a component of focal adhesion, which participates in various biological processes including cell migration, proliferation, and survival. ILK binds with pinch and parvin to form the ILK/pinch/parvin complex (IPP), thereby helping kindlin-2 activate αIIbβ3 [77]. Additionally, pinch participates in the modulation of chondrogenesis and bone mass, thus maintaining bone homeostasis [78]. ILK-deficient mice or cells are defective in ligand-binding, granule secretion, and platelet aggregation [79, 80].

Calcium and integrin-binding protein 1 (CIB1) is an αIIb-binding protein participating in c-Src activation. CIB1 is indispensable for platelet spreading [81] and CIB1^−/−^ mice are characterized by defective thrombosis [82]. Additionally, CIB1 is a regulator that enhances the activity of focal adhesion kinases and contributes to the formation of focal adhesions [83].

Vinculin is a focal adhesion protein with an autoinhibited structure and regulates mechanical coupling between the ECM and cytoskeleton [84]. The release of talin from autoinhibition causes a change in its conformation which allows vinculin to bind; this disrupts auto-inhibition and causes activation [85]. There is a PIP2 binding site in the vinculin tail and the PIP2/vinculin interaction results in a conformational change in vinculin, followed by vinculin activation. Vinculin-deficient mice are embryonic lethal [86].

Filamin (FLN) is a large actin-binding protein that contains three isoforms: FLNα, FLNb, and FLNc. *FLNa* mutations are associated with many diseases including skeletal dysplasia, neuronal migration abnormalities, and intestinal malrotation. FLNa mutations alter platelet production from the megakaryocytes, causing macrothrombocytopenia. In platelets per se, *FLNa* mutations may lead to impaired αIIbβ3 activation [87]. A recent study demonstrated that FLNα/αIIbβ3 interactions downregulate RhoA activity, which may have a significant positive effect on platelet count [88].

14–3-3ς is an integrin scaffolding adaptor which cooperates with c-Src and β3 to form the 14–3-3ς/c-Src/β3 complex. The binding site of 14–3-3ς to β3 is on the highly conserved EL17 motif [89]. Destruction of 14–3-3ς/c-Src/β3 complex inhibits thrombosis without influencing hemostasis [89]. The inhibitor of 14–3-3ς, 3′,4′,7′-trihydroxyisoflavone, is a potential antithrombotic drug, without the side effects of severe bleeding [89]. Meanwhile, 14–3-3ς interacts with GPIb-IX, contributing to the binding of vWF to GPIb-IX, thus supporting αIIbβ3 activation [90].

Profilin 1 is a small actin-binding protein that is essential for actin rearrangements. Platelets lacking profilin 1 are characterized by integrin inactivation, impaired platelet function, and microthrombocytopenia [91].

ADAP also attends to αIIbβ3 activation, stable adhesion, and cytoskeletal reorganization [92]. Under shear flow, ADAP^−/−^ mice fail to form stable thrombus in response to injury. ADAP depleted mice display an increased rebleeding time after tail cutting [92]. In addition, ADAP participates in platelet GPIb-IX-V signaling, which contributes to hemostasis and enhances the affinity of platelets for fibrinogen [93, 94]. ADAP helps talin and kindlin-3 transfer to the β3 cytoplasmic tail to support αIIbβ3 activation [95]. However, it is unclear whether ADAP directly binds to Talin and kindlin or not.

Smad4 is a transcription factor associated with transforming growth factor-beta induced integrin transcription and αIIbβ3 signal transduction. Smad4-deficient platelets display damaged functions, including platelet aggregation and spreading, fibrinogen binding and α-granule secretion, as well as clot retraction. Smad4^−/−^ mice suffer from mild thrombocytopenia, impaired platelet aggregation, and prolonged bleeding time [96].

Many members of the protein disulfide isomerase (PDI) family (including ERp5, ERp57, ERp72 and ERp46) are required for integrin activation and platelet function. Mice depleted in these proteins have impaired platelet aggregation and defective thrombosis [97, 98].

Calcium- and DAG-regulated guanine nucleotide exchange factor I (CalDAG-GEFI) is associated with Rap1 activity, integrin activation, and leukocyte adhesion. CalDAG-GEFI^−/−^ mice have increased bleeding time, impaired platelet aggregation, decreased thrombus formation, and neutrophil dysfunction. A 16-year-old girl with CalDAG-GEFI deficiency displays delayed αIIbβ3 activation, prolonged bleeding time, and impaired platelet granule release [99, 100]. Moreover, patients with a mutation in *RASGRP2,* encoding CalDAG-GEFI, demonstrate severe bleeding tendencies [101].

C-type lectin-like receptor 2 (CLEC-2) is considered a regulator of αIIbβ3 activation triggered by vWF binding to GPIbα in patients with thrombotic thrombocytopenic purpura (TTP). The hallmark of TTP is microvascular thrombosis. However, platelets lacking CLEC-2 reduce the formation of thrombosis by directly interacting with the extracellular domain of GPIbα [102].

Apoptosis signal-regulating kinase 1 (ASK1) is a member of the mitogen-activated protein kinase family. Ask1^−/−^ platelets have defects in integrin αIIbβ3 activation and aggregation and show a severe reduction in granule secretion [103].

##### Proteins which inhibit αIIbβ3 activation

SHANK-associated RH domain interactor (SHARPIN) acts as a suppressor during integrin activation by directly interacting with the αIIb subunit and blocking the recruitment of talin to the cytoplasmic tail [104]. SHARPIN-null mouse platelets exhibit increased spreading on fibrinogen, increased soluble fibrinogen binding in response to sub-maximal ADP concentrations with an increased colocalization of αIIbβ3 with talin [104]. However, SHARPIN-null mice show impaired thrombus growth and slightly prolonged tail bleeding time [104]. The V^267^ and L^276^ residues of SHARPIN play a vital role in SHARPIN-mediated inhibition of αIIbβ3 [105]. SHARPIN is also associated with tumor proliferation, migration, and invasion. A recent study showed that SHARPIN negatively impacts melanoma prognosis by upregulating Rap1 [105].

Twinfilin 2a (Twf2a) is a small actin-binding protein and is indispensable for suppression of cytoskeletal dynamics by inhibiting the activity of cofilin and profilin 1. As shown above, talin is the final step of integrin activation. Talin and β3 colocalization increases in Twf2a^−/−^ mice. Twf2a^−/−^ mice display accelerated arterial thrombus formation, shorter tail bleeding time, and mild macrothrombocytopenia [106]. In contrast, twinfilin1 deficient mice have no apparent change in platelet function. However, deficiencies in twinfilin1 and cofilin1 result in severe macrothrombocytopenia and impaired platelet function [107].

Thioredoxin-related transmembrane protein 1 (TMX1) is a member of the polydispersity index family associated with platelet function. TMX1 exerts a negative influence on platelet function which contrasts with the positive regulation of platelet function by the other polydispersity index members, such as ERp5, ERp57, and ERp72 [97, 108, 109]. TMX1 inhibits platelet aggregation, ATP release, and αIIbβ3 activation [106, 108].

Downstream of the kinase (DOK) negatively affects αIIbβ3 activation by competing with talin [110]. Clot retraction is impaired in Dok1^−/−^ mice [111]. DOK2 directly interacts with the NPxY^747^ motif of β3, while DOK1 does not [112]. Additionally, DOK2 is responsible for the adhesion function of integrin αIIbβ3. Dok1^−/−^ mice are characterized by enhanced platelet aggregation and thrombus formation [113]. DOK1 forms a complex with 14–3-3ς via the phosphotyrosine-binding domain which interacts with the β3 cytoplasmic tail to regulate integrin αIIbβ3 bidirectional signaling [114].

Adenosine 5-diphosphate-ribosylation factors (Arfs) are small guanosine triphosphate-binding proteins that regulate endocytic trafficking, actin cytoskeleton remodeling, and lipid metabolism. *Arf-6* knockout mice have enhanced platelet spreading on fibrinogen and faster clot retraction. This may be not the result of alterations in αIIbβ3 signaling because myosin light-chain phosphorylation and Rac1/RhoA activation are unaffected [115].

Other proteins also inhibit platelet function, such as CEACAM1/2, PECAM-1, CLP36, c-Cbl, PKC delta, PLD2, and so on [116]. Although deeper understanding of the proteins interacting with αIIbβ3 was explored, the exact mechanism of its activation and the switch point of bidirectional signal transduction have not been clarified fully. The purpose of exploring the mechanism is to develop safer clinical treatment. At present, αIIbβ3 has been confirmed to have prospects in thrombosis, hemostasis and tumor treatment. It is known to all that the understanding of integrin bidirectional signal is far from complete. High-resolution imaging, proteomics and structural biology should be highly valued in further research.

Above interacting factors are shown in Table 1.

**Table 1:** Proteins associated with the connection of ECM to the cytoskeleton

Proteins	Function	Phenotype of knockout mice	reference
Talin	support integrin activation, link ECM and cytoskeleton	impaired platelet aggregation and adhesion, spontaneous hemorrhage and pathological bleeding	[117]
Kindlin-3	support integrin activation, platelet aggregation, platelet adhesion	severe bleeding and resistance to arterial thrombosis	[46]
Paxillin	support αIIbβ3 activation, influence focal adhesion	delayed spreading, impaired migration	[118]
Vinculin	regulate mechanical coupling, cell adhesion, control focal adhesion formation	normal fibrinogen binding, aggregation, spreading, actin organization, clot retraction, prolonged bleeding time	[119]
Rap1	stimulate integrin activation	impaired spreading on fibrinogen, prolonged tail bleeding time, and reduced platelet aggregation.	[30]
Filamin	modulate actin crosslinking, cell migration	increased tail bleeding time, macrothrombocytopenia	[88, 120]
ADAP	support αIIbβ3 activation, stable adhesion, cytoskeletal reorganization	shorter life span, microthrombocytopenia, impaired cytoskeletal reorganization, and thrombus formation	[93] [92]
ILK	support αIIbβ3 activation	impaired ligand-binding, granule secretion, and platelets aggregation	[79, 80]
Profilin	regulate actin rearrangement	microthrombocytopenia, accelerated integrin inactivation	[91]
Cofilin	promote F-actin assembly, thrombus stabilization, actin organization	microthrombocytopenia, unstable adhesion	[93, 94]
Twf2a	suppress cytoskeletal dynamics	accelerated arterial thrombus formation, faster tail bleeding time, and mild macrothrombocytopenia	[106]
SHARPIN	inhibit integrin activation	increased binding of fibrinogen to αIIbβ3 and impaired thrombus formation	[104]
actinin	trigger adhesion maturation	enhance force generation, impaired mechanotransduction	[121]
RhoA	coordinate changes in the actin cytoskeleton, actin stress fiber formation	defective platelet production and marked thrombocytopenia	[122]
Cdc42	facilitate filopodial protrusions and lamellipodia formation, cytoskeletal rearrangements	severe thrombocytopenia, macrothrombocytopenia, abnormal platelet morphology, impaired platelet function	[123, 124]
Rac1			
Arf6	regulate endocytic trafficking, actin cytoskeleton remodeling, and lipid metabolism	enhanced spreading on fibrinogen and clot retraction	[115]

### Integrin αIIbβ3 and shear stress

Under shear conditions, GPIb-IX-V/vWF interaction is regarded as the main contributor for capture and arrest of flowing platelets at injured sites of vessels, and for initiation of platelet adhesion [125]. While, αIIbβ3 is responsible for platelet stable adhesion and required to resist the washing or shedding by shear stress [56, 126–128], contributing to maintain the attachment-detachment balance of thrombus formation under flow [129]. β3-knockout or β3-blocked platelets hardly adhere and aggregate to the wound or wound-mimicking surface at shear stress in vivo [58] or in vitro [129, 130]. Under flow in vivo, the thrombus formed in the mice with β3 double mutations in Y^747^ and Y^759^, which have a defect in αIIbβ3 outside-in signaling, have a loosely-packed and less-activated core, compared that in wild-type mice [131]. High supraphysiologic shear stress can induce αIIbβ3 activation, but also shed αIIbβ3 from platelet surface [132]. Recently, it is found that shear stress can downregulate platelet αIIbβ3 via redistribution to αIIbβ3-enriched microparticles, rather than via enzymatic shedding of their ectodomains, and sheared platelets show reduced levels of activated αIIbβ3 and lower aggregation under the stimulation by biochemical agonists [133].

### Integrin αIIbβ3 and tumor

Platelet-cancer interaction has been recognized for a long time. The tumor cells contribute to platelet activation, adhesion and aggregation. Conversely, activated platelets can interact with tumor cells facilitating tumor progression, migration, and growth [134, 135]. Under surgery, a main treatment for solid tumor, platelets adopt a TLR4-dependent manner to aggregate circulating tumor cells and lead to distant metastasis through ERK5-αIIbβ3 signaling [136]. Antiplatelet drugs contribute to tumor prevention and treatment [137, 138]. After treatment with αIIbβ3 antagonist, abciximab, the release of platelet-derived vascular endothelial growth factor and the adhesion of platelets to tumor cells are significantly impaired, restricting the extravasation of tumor cells, thus inhibiting metastasis [139]. Another study of eptifibatide, another αIIbβ3 antagonist, also confirms the role of αIIbβ3 in adhesion and hematogenous metastasis of breast cancer [140]. The oral αIIbβ3 inhibitor, XV454, inhibits lung metastasis of tumor cells [141]. Cadherin 6 (CDH6), highly expressed in renal cancer and ovarian cancer, interplays with αIIbβ3 via its RGD sequence, regulating tumor adhesion, migration and invasion [142]. These functions can be inhibited by RGD-based monoclonal antibodies or eptifibatide [142]. 1-(4-isopropyl-phenyl)-β-carboline-3-carboxylic acid (ICCA) is designed to inhibit both αIIbβ3 and P-selectin and exerts a negative role in tumor growth and thrombosis [143, 144]. Additionally, some tumor cells, such as breast cancer, melanoma, or lung carcinoma, also ectopically express αIIbβ3, besides αvβ3 [145]. The αIIbβ3-expressing tumor cell lines and the role of αIIbβ3 in them are listed in Table 2**.**

**Table 2:** Integrin αIIbβ3 in tumor cell lines

Type of tumors	Cell lines	αIIbβ3 function in tumors	References
Breast cancer	MDA-MB-231	facilitate cell adhesion, migration and invasion	[140, 146]
Melanoma	A375	promote adhesion	[139]
	B16	promote adhesion, and metastasis	[147]
	M3Dau	induce formation of platelet-tumor aggregates	[148]
Lewis lung carcinoma	LL2	facilitate metastasis	[139]
Renal cancer	786-O	facilitate adhesion, migration, proliferation and invasion	[142]
Colon carcinoma	CT26	induce tumor cells binding to platelets	[149]
Ovarian cancer	SKOV-3	facilitate adhesion, migration, proliferation and invasion	[142]

### Novel inhibitors target αIIbβ3

αIIbβ3 has become a validated target for inhibiting aberrant platelet aggregation in light of its important role in platelet activation and function. Many oral αIIbβ3 antagonists, such as orbofiban, sibrafiban, xemilofiban, lotrafiban and roxifiban, have been tested, but clinical trials were stopped due to prolonged bleeding time, increased incidence of thrombocytopenia, and growing mortality [150, 151]. To date, only three drugs are approved by FDA to use in humans, mainly in percutaneous coronary intervention (PCI) to improve prognosis and reduce mortality in patients with acute myocardial infarction [152], including abciximab, eptifibatide, and tirofiban. Recently, some novel agents based on integrin have been developed. All of the antagonists list above are characterized by affecting hemostasis and lead to a risk of bleeding or prolonged bleeding time. In addition, intravenous injection limits their clinical application when they display their antiplatelet function [150]. This enlightens us to determine the switch that exhibits anti-platelet activity without affecting hemostasis. Some new targets have been found based on αIIbβ3, compared with antagonists above，they have made improvements in reducing bleeding side effects and inhibiting integrin conformational changes. RUC-4 is a novel αIIbβ3 antagonist that plays a key role in blocking platelet aggregation, thrombosis, and αIIbβ3-fibrinogen binding [153]. RUC-4 is more effective and soluble, and shows greater specificity for integrin αIIbβ3 [154]. RUC-4 was first used in 2020 on patients with coronary artery disease; it demonstrated good inhibition of platelet aggregation with no serious side effects [153]. What’s more, RUC-4 does not induce integrin extension [154].

Hr10 is a peptide designed based on the RGD sequence. Unlike tirofiban, Hr10 has no effect on the conformational changes of αIIbβ3. Mice treated with Hr10 show inhibited thrombosis, while without serious bleeding. A modified tirofiban serving as a “pure” antagonist disrupts the interaction between fibrinogen and αIIbβ3 to negatively affect platelet aggregation. Notably, it has no influence on clot contraction; thus, the hemostatic function is preserved [155]. However, a recent study confirms that clot retraction and platelet aggregation can be inhibited by modified tirofiban at an appropriate concentration. Contrary to previous conclusions, M-tirofiban can enhance ligand-induced binding sites exposure and induce conformational changes [156].

TMV-7 is another type of disintegrin isolated from snake venom. TMV-7 selectively inhibits the outside-in signal transduction mediated by Gα13, but does not affect the inside-out signal. The binding site of TMV-7 is located between the αIIb and β3 subunits around the β-propeller, and its binding does not lead to conformational changes in αIIbβ3 [157].

ANTP266 plays a pivotal role in αIIbβ3 inhibition. It has a short plasma half-life and is used to treat emergent heart diseases, such as acute myocardial infarction without increased bleeding time [158].

Dieckol, a phlorotannin, can reduce thrombosis and induce fibrinolysis [159, 160]. Dieckol suppresses αIIbβ3 activation through cAMP-PKA-VASP pathway, thereby impairing platelet aggregation and granule release, without severe bleeding risk [161].

For a more in-depth learning of integrin αIIbβ3 antagonists, several comprehensive reviews have been selected for further reading [5, 150, 162].

## Conclusion

An increasing number of people die of cardiovascular thrombotic diseases each year, mainly caused by abnormal platelet activation. Integrin αIIbβ3 is the most abundant receptor in platelets and plays a key role in platelet activation. Recently, a deeper understanding of the internal mechanism of αIIbβ3 activation and more interactive proteins are discovered. However, the interactions between αIIbβ3 and related proteins are too complex to elucidate. The goal of understanding αIIbβ3 activation is to identify new targets for the development of safe antithrombotic and antitumor drugs. Further research is needed to develop an efficient antiplatelet drug that does not alter hemostasis.

## Data Availability

Not applicable.

## Abbreviations

ADP: Adenosine diphosphate
ADAP: Adhesion and degranulation promoting adapter protein
Arf: Adenosine 5-diphosphate-ribosylation factors
Ask1: Apoptosis signal-regulating kinase 1
βTD: β tail domain
CIB1: Calcium- and integrin-binding protein 1
CalDAG-GEFI: Ca2^2+^ and DAG-regulated guanine nucleotide exchange factor I
CLEC-2: C-type lectin-like receptor 2
Csk: c-Src kinase
DOK: Down-stream of tyrosine kinase
EGF: Epidermal growth factor
ECM: Extracellular matrix
FLN: Filamin
FDA: Food and Drug Administration
FERM: band 4.1, ezrin, radixin, moesin
HPA: Human platelet antigen
IMC: Inner membrane clasp
IBS: Integrin binding site
ILK: Integrin-linked kinase
ICAP-1: Integrin cytoplasmic domain associated protein-1
JAM-A: Junctional adhesion molecule A
OMC: Outer membrane clasp
PDI: Protein disulfide isomerase
PH: Pleckstrin homology
PSI: Plexin-sempahorin-integrin
PIPK1γ: Phosphatidyl inositol phosphate kinase type 1γ
PIP2: Phosphotidylinositol 4,5-bis- phosphate
PCI: Percutaneous coronary intervention
PI3Ks: Phosphoinositide 3-kinases
RIAM: Rap1-GTP-interacting adaptor molecule
SHARPIN: Shank-associated RH domain interacting protein
SFK: Src family kinase
Syk: Spleen tyrosine kinase
TMX1: Thioredoxin-related transmembrane protein 1
Talin-H: N-terminal head domain of talin
Talin-R: C-terminal flexible rod domain of talin
Twf2a: Twinfilin 2a
VBS: Vinculin binding site
